# Supplementary material for: Computational Repurposing of Mitoxantrone-Related Structures against Monkeypox Virus: A Molecular Docking and 3D Pharmacophore Study
Source: Int J Mol Sci. 2022 Nov 18;23(22):14287. doi: 10.3390/ijms232214287 (PMC9695275; doi:10.3390/ijms232214287)
Supplement: Supplementary file 1 [file ijms-23-14287-s001.zip › ijms-1968805-supplementary.pdf]

International Journal of *Molecular Sciences*

MDPI

Table S1: Anthracene compounds used in the study.

Compounds	Pubchem ID
**MXN** (Standard)	4212
**1**	13276605
**2**	153640723
**3**	44275839
**4**	71044822
**5**	142963046
**6**	44316536
**7**	24848320
**8**	59835539
**9**	44541200
**10**	58102019
**11**	143270488
**12**	145293737
**13**	44541201

Table S2: Predicted binding energy values of ligands with receptor 2V54

Receptor filename	Ligand filename	ligand's mode	Affinity (kcal/mol)	Ki (μmol)	Lower bound of the RMSD from this ligand's best mode (A)	Upper bound of the RMSD from this ligand's best mode (A)
2v54clean	13276605	1	-7.2	5.27672	0	0
2v54clean	13276605	2	-7.2	5.27672	2.677	3.252
2v54clean	13276605	3	-7.1	6.2469	2.588	6.787
2v54clean	13276605	4	-6.9	8.75519	3.283	6.402
2v54clean	13276605	5	-6.6	14.5267	2.126	2.528
2v54clean	13276605	6	-6.6	14.5267	2.016	3.182
2v54clean	13276605	7	-6.5	17.1976	2.752	4.248
2v54clean	13276605	8	-6.3	24.1028	3.557	6.578
2v54clean	13276605	9	-6.1	33.7808	2.162	5.848
2v54clean	13276605	10	-6	39.9917	2.721	5.199
2v54clean	142963046	1	-6.9	8.75519	0	0
2v54clean	142963046	2	-6.6	14.5267	1.988	3.195
2v54clean	142963046	3	-6.6	14.5267	1.125	6.218
2v54clean	142963046	4	-6.3	24.1028	2.895	6.84
2v54clean	142963046	5	-6	39.9917	3.1	7.356
2v54clean	142963046	6	-5.8	56.0494	3.15	5.982
2v54clean	142963046	7	-5.7	66.3546	2.019	2.62

2v54clean	142963046	8	-5.3	130.339	3.896	7.486
2v54clean	142963046	9	-5.2	154.303	1.752	2.193
2v54clean	142963046	10	-5.1	182.673	3.399	6.191
2v54clean	143270488	1	-7.2	5.27672	0	0
2v54clean	143270488	2	-6.5	17.1976	2.385	5.698
2v54clean	143270488	3	-6.4	20.3595	2.22	6.328
2v54clean	143270488	4	-6.3	24.1028	3.158	6.25
2v54clean	143270488	5	-6.2	28.5344	3.037	6.178
2v54clean	143270488	6	-6.2	28.5344	2.742	6.175
2v54clean	143270488	7	-6.2	28.5344	2.284	3.782
2v54clean	143270488	8	-6.1	33.7808	2.923	6.619
2v54clean	143270488	9	-5.7	66.3546	3.413	7.671
2v54clean	143270488	10	-5.7	66.3546	2.617	5.779
2v54clean	145293737	1	-7	7.39545	0	0
2v54clean	145293737	2	-6.9	8.75519	1.943	3.237
2v54clean	145293737	3	-6.8	10.3649	2.85	7.409
2v54clean	145293737	4	-6.8	10.3649	2.372	6.88
2v54clean	145293737	5	-6.8	10.3649	1.882	2.153
2v54clean	145293737	6	-6.7	12.2706	3.038	5.894
2v54clean	145293737	7	-6.5	17.1976	2.939	6.151
2v54clean	145293737	8	-6.4	20.3595	2.59	5.254
2v54clean	145293737	9	-6.3	24.1028	2.861	6.372
2v54clean	145293737	10	-6.2	28.5344	2.896	7.459
2v54clean	153640723	1	-7.2	5.27672	0	0
2v54clean	153640723	2	-7.2	5.27672	2.668	3.286
2v54clean	153640723	3	-7.1	6.2469	2.367	5.966
2v54clean	153640723	4	-6.8	10.3649	3.716	6.539
2v54clean	153640723	5	-6.7	12.2706	3.187	5.325
2v54clean	153640723	6	-6.6	14.5267	2.69	4.803
2v54clean	153640723	7	-6.5	17.1976	2.493	4.191
2v54clean	153640723	8	-6.5	17.1976	2.637	4.164
2v54clean	153640723	9	-6.3	24.1028	3.261	4.329
2v54clean	153640723	10	-6.3	24.1028	3.064	6.137
2v54clean	24848320	1	-6.9	8.75519	0	0
2v54clean	24848320	2	-6.7	12.2706	0.812	6.656
2v54clean	24848320	3	-6.6	14.5267	1.845	2.134
2v54clean	24848320	4	-6.5	17.1976	3.032	6.141
2v54clean	24848320	5	-6.5	17.1976	1.82	6.33
2v54clean	24848320	6	-6.5	17.1976	2.945	5.989
2v54clean	24848320	7	-6.4	20.3595	2.945	6.527
2v54clean	24848320	8	-6.3	24.1028	3.08	7.142

2v54clean	24848320	9	-6.2	28.5344	2.554	5.307
2v54clean	24848320	10	-6.2	28.5344	2.267	5.936
2v54clean	4212	1	-6.9	8.75519	0	0
2v54clean	4212	2	-6.9	8.75519	0.578	6.115
2v54clean	4212	3	-6.8	10.3649	1.809	3.049
2v54clean	4212	4	-6.4	20.3595	2.901	7.229
2v54clean	4212	5	-6.4	20.3595	3.038	6.124
2v54clean	4212	6	-6.2	28.5344	1.982	6.079
2v54clean	4212	7	-6.2	28.5344	2.113	5.751
2v54clean	4212	8	-5.7	66.3546	3.824	7.386
2v54clean	4212	9	-5.7	66.3546	3.121	5.983
2v54clean	4212	10	-5.6	78.5546	3.637	5.924
2v54clean	44275839	1	-6.7	12.2706	0	0
2v54clean	44275839	2	-6.7	12.2706	0.9	6.096
2v54clean	44275839	3	-6.5	17.1976	2.859	5.833
2v54clean	44275839	4	-6.5	17.1976	2.911	5.818
2v54clean	44275839	5	-6.4	20.3595	1.517	5.892
2v54clean	44275839	6	-6.4	20.3595	3.004	7.282
2v54clean	44275839	7	-6.3	24.1028	2.457	5.907
2v54clean	44275839	8	-6.2	28.5344	2.088	3.222
2v54clean	44275839	9	-6.2	28.5344	2.961	5.788
2v54clean	44275839	10	-6.1	33.7808	2.447	6.669
2v54clean	44316536	1	-6.8	10.3649	0	0
2v54clean	44316536	2	-6.7	12.2706	1.813	3.034
2v54clean	44316536	3	-6.5	17.1976	1.231	5.944
2v54clean	44316536	4	-6.2	28.5344	2.226	5.584
2v54clean	44316536	5	-6.1	33.7808	1.901	6.001
2v54clean	44316536	6	-6	39.9917	1.726	5.318
2v54clean	44316536	7	-6	39.9917	2.733	5.979
2v54clean	44316536	8	-6	39.9917	2.912	6.556
2v54clean	44316536	9	-5.8	56.0494	2.411	5.181
2v54clean	44316536	10	-5.7	66.3546	2.968	7.503
2v54clean	44541200	1	-7	7.39545	0	0
2v54clean	44541200	2	-6.9	8.75519	1.918	3.139
2v54clean	44541200	3	-6.9	8.75519	2.252	3.746
2v54clean	44541200	4	-6.5	17.1976	1.953	5.693
2v54clean	44541200	5	-6.4	20.3595	3.924	6.984
2v54clean	44541200	6	-6.3	24.1028	1.496	2.173
2v54clean	44541200	7	-6.3	24.1028	3.802	7.497
2v54clean	44541200	8	-6.2	28.5344	3.564	6.246
2v54clean	44541200	9	-6	39.9917	2.437	6.312

2v54clean	44541200	10	-5.9	47.3446	2.131	6.524
2v54clean	44541201	1	-6.8	10.3649	0	0
2v54clean	44541201	2	-6.8	10.3649	2.074	6.649
2v54clean	44541201	3	-6.8	10.3649	2.119	3.212
2v54clean	44541201	4	-6.6	14.5267	1.917	6.639
2v54clean	44541201	5	-6.5	17.1976	2.169	4.777
2v54clean	44541201	6	-6.4	20.3595	3.751	7.201
2v54clean	44541201	7	-6.2	28.5344	2.043	4.629
2v54clean	44541201	8	-6.1	33.7808	2.609	4.817
2v54clean	44541201	9	-6.1	33.7808	2.497	5.153
2v54clean	44541201	10	-5.9	47.3446	4.149	8.014
2v54clean	58102019	1	-7	7.39545	0	0
2v54clean	58102019	2	-6.7	12.2706	1.911	5.965
2v54clean	58102019	3	-6.6	14.5267	2.359	3.624
2v54clean	58102019	4	-6.5	17.1976	1.615	6.131
2v54clean	58102019	5	-6.4	20.3595	2.534	6.456
2v54clean	58102019	6	-6.4	20.3595	2.623	5.977
2v54clean	58102019	7	-6.3	24.1028	2.647	7.223
2v54clean	58102019	8	-5.6	78.5546	3.546	7.071
2v54clean	58102019	9	-5.4	110.096	2.705	7.76
2v54clean	58102019	10	-5.4	110.096	4.009	7.755
2v54clean	59835539	1	-7.2	5.27672	0	0
2v54clean	59835539	2	-7.1	6.2469	1.836	3.047
2v54clean	59835539	3	-6.5	17.1976	2.313	4.97
2v54clean	59835539	4	-6.4	20.3595	1.404	6.049
2v54clean	59835539	5	-6.3	24.1028	3.213	6.851
2v54clean	59835539	6	-6.2	28.5344	3.153	7.713
2v54clean	59835539	7	-5.8	56.0494	2.537	5.137
2v54clean	59835539	8	-5.6	78.5546	2.015	6.715
2v54clean	59835539	9	-5.6	78.5546	3.842	6.376
2v54clean	59835539	10	-5.6	78.5546	2.212	2.802
2v54clean	71044822	1	-6.9	8.75519	0	0
2v54clean	71044822	2	-6.7	12.2706	1.8	3.092
2v54clean	71044822	3	-6.5	17.1976	1.474	1.709
2v54clean	71044822	4	-6.3	24.1028	1.915	2.9
2v54clean	71044822	5	-6.3	24.1028	3.097	7.205
2v54clean	71044822	6	-6.2	28.5344	2.369	3.57
2v54clean	71044822	7	-6.2	28.5344	2.831	5.741
2v54clean	71044822	8	-6.2	28.5344	1.885	6.342
2v54clean	71044822	9	-6	39.9917	2.45	5.247
2v54clean	71044822	10	-5.8	56.0494	2.413	3.05

Table S3: Predicted binding energy values of ligands with receptor 4QWO

Receptor filename	Ligand filename	Ligand's mode	Affinity (kcal/mol)	Ki (µmol)	Lower bound of the RMSD from this ligand's best mode (A)	Upper bound of the RMSD from this ligand's best mode (A)
4qwoclean	13276605	1	-6.6	14.527	0	0
4qwoclean	13276605	2	-6.5	17.198	2.675	6.047
4qwoclean	13276605	3	-6.4	20.36	3.149	6.756
4qwoclean	13276605	4	-6.3	24.103	2.116	2.7
4qwoclean	13276605	5	-6.2	28.534	1.895	5.253
4qwoclean	13276605	6	-6.2	28.534	3.207	5.849
4qwoclean	13276605	7	-6.2	28.534	2.468	6.045
4qwoclean	13276605	8	-6.1	33.781	2.965	7.019
4qwoclean	13276605	9	-6.1	33.781	2.369	6.247
4qwoclean	13276605	10	-6	39.992	3.238	7.156
4qwoclean	142963046	1	-6.1	33.781	0	0
4qwoclean	142963046	2	-6	39.992	2.572	5.116
4qwoclean	142963046	3	-6	39.992	2.702	5.052
4qwoclean	142963046	4	-6	39.992	2.524	6.237
4qwoclean	142963046	5	-5.9	47.345	3.107	7.056
4qwoclean	142963046	6	-5.9	47.345	3.239	6.969
4qwoclean	142963046	7	-5.9	47.345	1.549	2.06
4qwoclean	142963046	8	-5.9	47.345	1.529	6.599
4qwoclean	142963046	9	-5.7	66.355	2.832	5.336
4qwoclean	142963046	10	-5.7	66.355	2.706	6.313
4qwoclean	143270488	1	-6.6	14.527	0	0
4qwoclean	143270488	2	-6.5	17.198	2.603	5.344
4qwoclean	143270488	3	-6.3	24.103	2.402	5.705
4qwoclean	143270488	4	-6.3	24.103	0.934	6.79
4qwoclean	143270488	5	-6.2	28.534	3.04	5.832
4qwoclean	143270488	6	-6.2	28.534	2.563	5.592
4qwoclean	143270488	7	-6.2	28.534	2.823	7.505
4qwoclean	143270488	8	-6.1	33.781	2.487	6.982
4qwoclean	143270488	9	-6.1	33.781	2.868	5.844
4qwoclean	143270488	10	-6	39.992	2.694	7.227
4qwoclean	145293737	1	-6.8	10.365	0	0
4qwoclean	145293737	2	-6.6	14.527	1.799	2.494
4qwoclean	145293737	3	-6.6	14.527	2.545	7.626
4qwoclean	145293737	4	-6.5	17.198	1.535	6.839
4qwoclean	145293737	5	-6.5	17.198	1.594	2.025

4qwoclean	145293737	6	-6.4	20.36	2.451	5.163
4qwoclean	145293737	7	-6.2	28.534	1.677	6.891
4qwoclean	145293737	8	-6.2	28.534	2.551	5.965
4qwoclean	145293737	9	-6.1	33.781	3.192	5.815
4qwoclean	145293737	10	-6.1	33.781	2.582	5.096
4qwoclean	153640723	1	-6.8	10.365	0	0
4qwoclean	153640723	2	-6.7	12.271	2.553	5.836
4qwoclean	153640723	3	-6.7	12.271	1.692	2.264
4qwoclean	153640723	4	-6.7	12.271	2.804	6.861
4qwoclean	153640723	5	-6.6	14.527	1.678	2.033
4qwoclean	153640723	6	-6.6	14.527	2.545	5.908
4qwoclean	153640723	7	-6.5	17.198	2.49	5.493
4qwoclean	153640723	8	-6.3	24.103	2.529	4.713
4qwoclean	153640723	9	-6.2	28.534	1.785	4.836
4qwoclean	153640723	10	-6.2	28.534	2.8	4.808
4qwoclean	24848320	1	-6.8	10.365	0	0
4qwoclean	24848320	2	-6.7	12.271	1.45	1.944
4qwoclean	24848320	3	-6.7	12.271	1.346	6.793
4qwoclean	24848320	4	-6.6	14.527	3.278	7.963
4qwoclean	24848320	5	-6.6	14.527	2.78	7.755
4qwoclean	24848320	6	-6.6	14.527	2.734	5.438
4qwoclean	24848320	7	-6.5	17.198	2.77	7.045
4qwoclean	24848320	8	-6.5	17.198	2.726	5.307
4qwoclean	24848320	9	-6.5	17.198	3.155	5.504
4qwoclean	24848320	10	-6.3	24.103	3.103	7.342
4qwoclean	4212	1	-6.7	12.271	0	0
4qwoclean	4212	2	-6.7	12.271	1.597	2.061
4qwoclean	4212	3	-6.7	12.271	3.001	7.405
4qwoclean	4212	4	-6.7	12.271	1.295	6.805
4qwoclean	4212	5	-6.6	14.527	2.505	6.548
4qwoclean	4212	6	-6.5	17.198	3.002	5.424
4qwoclean	4212	7	-6.4	20.36	2.758	7
4qwoclean	4212	8	-6.3	24.103	2.675	7.391
4qwoclean	4212	9	-6.2	28.534	4.641	7.831
4qwoclean	4212	10	-6.1	33.781	2.639	5.371
4qwoclean	44275839	1	-6.4	20.36	0	0
4qwoclean	44275839	2	-6.4	20.36	3.07	5.366
4qwoclean	44275839	3	-6.2	28.534	1.921	2.423
4qwoclean	44275839	4	-6.2	28.534	2.238	3.169
4qwoclean	44275839	5	-6.2	28.534	0.749	6.356
4qwoclean	44275839	6	-6.2	28.534	3.082	7.377

4qwoclean	44275839	7	-6.1	33.781	2.991	5.533
4qwoclean	44275839	8	-6	39.992	1.824	6.062
4qwoclean	44275839	9	-5.9	47.345	2.201	6.07
4qwoclean	44275839	10	-5.9	47.345	2.464	7.08
4qwoclean	44316536	1	-6.2	28.534	0	0
4qwoclean	44316536	2	-6.1	33.781	2.781	6.844
4qwoclean	44316536	3	-6.1	33.781	2.517	6.309
4qwoclean	44316536	4	-6	39.992	2.819	5.463
4qwoclean	44316536	5	-6	39.992	2.445	4.9
4qwoclean	44316536	6	-5.9	47.345	2.479	6.695
4qwoclean	44316536	7	-5.9	47.345	2.981	7.543
4qwoclean	44316536	8	-5.9	47.345	2.736	5.218
4qwoclean	44316536	9	-5.7	66.355	2.456	6.76
4qwoclean	44316536	10	-5.7	66.355	1.414	6.715
4qwoclean	44541200	1	-6.1	33.781	0	0
4qwoclean	44541200	2	-6	39.992	2.718	5.637
4qwoclean	44541200	3	-6	39.992	1.557	2.152
4qwoclean	44541200	4	-5.9	47.345	2.811	7.379
4qwoclean	44541200	5	-5.9	47.345	1.032	1.12
4qwoclean	44541200	6	-5.9	47.345	2.069	3.154
4qwoclean	44541200	7	-5.8	56.049	2.196	6.993
4qwoclean	44541200	8	-5.7	66.355	1.949	3.068
4qwoclean	44541200	9	-5.7	66.355	2.002	6.387
4qwoclean	44541200	10	-5.6	78.555	2.541	3.58
4qwoclean	44541201	1	-6.3	24.103	0	0
4qwoclean	44541201	2	-5.9	47.345	3.134	5.885
4qwoclean	44541201	3	-5.8	56.049	1.414	2.362
4qwoclean	44541201	4	-5.8	56.049	2.285	5.267
4qwoclean	44541201	5	-5.8	56.049	2.823	5.726
4qwoclean	44541201	6	-5.8	56.049	2.574	6.062
4qwoclean	44541201	7	-5.7	66.355	2.581	4.984
4qwoclean	44541201	8	-5.7	66.355	2.85	5.657
4qwoclean	44541201	9	-5.7	66.355	2.632	5.546
4qwoclean	44541201	10	-5.7	66.355	2.228	5.201
4qwoclean	58102019	1	-6.8	10.365	0	0
4qwoclean	58102019	2	-6.7	12.271	3.07	5.589
4qwoclean	58102019	3	-6.6	14.527	1.366	7.103
4qwoclean	58102019	4	-6.6	14.527	2.551	4.971
4qwoclean	58102019	5	-6.6	14.527	2.868	5.507
4qwoclean	58102019	6	-6.5	17.198	2.781	5.145
4qwoclean	58102019	7	-6.4	20.36	2.982	7.803

4qwoclean	58102019	8	-6.3	24.103	3.09	6.172
4qwoclean	58102019	9	-6.3	24.103	2.64	5.023
4qwoclean	58102019	10	-6.3	24.103	2.306	5.422
4qwoclean	59835539	1	-6	39.992	0	0
4qwoclean	59835539	2	-6	39.992	2.604	5.095
4qwoclean	59835539	3	-5.9	47.345	2.798	6.999
4qwoclean	59835539	4	-5.9	47.345	1.834	2.521
4qwoclean	59835539	5	-5.8	56.049	2.759	6.28
4qwoclean	59835539	6	-5.7	66.355	1.353	5.92
4qwoclean	59835539	7	-5.7	66.355	1.797	2.299
4qwoclean	59835539	8	-5.6	78.555	1.523	2.419
4qwoclean	59835539	9	-5.6	78.555	2.16	6.614
4qwoclean	59835539	10	-5.6	78.555	1.56	6.28
4qwoclean	71044822	1	-6.6	14.527	0	0
4qwoclean	71044822	2	-6.5	17.198	2.666	5.224
4qwoclean	71044822	3	-6.4	20.36	2.627	5.267
4qwoclean	71044822	4	-6.4	20.36	2.946	5.3
4qwoclean	71044822	5	-6.3	24.103	3.119	7.771
4qwoclean	71044822	6	-6.3	24.103	2.653	6.691
4qwoclean	71044822	7	-6.2	28.534	2.624	5.952
4qwoclean	71044822	8	-6.1	33.781	1.822	6.799
4qwoclean	71044822	9	-6	39.992	2.898	6.583
4qwoclean	71044822	10	-5.9	47.345	2.301	6.061

Table S4: Predicted binding energy values of ligands with receptor 6BED

Receptor filename	Ligand filename	Ligand's mode	Affinity (kcal/mol)	Ki (μmol)	Lower bound of the RMSD from this ligand's best mode (A)	Upper bound of the RMSD from this ligand's best mode (A)
6bedclean	13276605	1	-7.9	1.6191	0	0
6bedclean	13276605	2	-7.9	1.6191	1.711	2.455
6bedclean	13276605	3	-7.9	1.6191	2.991	6.774
6bedclean	13276605	4	-7.9	1.6191	2.53	5.875
6bedclean	13276605	5	-7.7	2.2691	2.321	5.61
6bedclean	13276605	6	-7.4	3.765	1.903	2.139
6bedclean	13276605	7	-7.3	4.4572	2.611	5.203
6bedclean	13276605	8	-7.3	4.4572	2.275	4.107
6bedclean	13276605	9	-7.3	4.4572	2.434	5.215
6bedclean	13276605	10	-7.3	4.4572	2.662	6.347

6bedclean	142963046	1	-7.3	4.4572	0	0
6bedclean	142963046	2	-7.2	5.2767	2.856	3.834
6bedclean	142963046	3	-7.2	5.2767	3.977	5.749
6bedclean	142963046	4	-7	7.3955	2.776	5.999
6bedclean	142963046	5	-6.9	8.7552	2.961	7.314
6bedclean	142963046	6	-6.8	10.365	3.411	7.39
6bedclean	142963046	7	-6.7	12.271	4.044	4.856
6bedclean	142963046	8	-6.7	12.271	3.46	5.718
6bedclean	142963046	9	-6.4	20.36	16.72	20.524
6bedclean	142963046	10	-6.3	24.103	3.152	4.219
6bedclean	143270488	1	-7.4	3.765	0	0
6bedclean	143270488	2	-7.2	5.2767	2.968	5.397
6bedclean	143270488	3	-7	7.3955	3.398	6.282
6bedclean	143270488	4	-7	7.3955	2.975	6.736
6bedclean	143270488	5	-6.9	8.7552	2.935	6.771
6bedclean	143270488	6	-6.9	8.7552	1.421	1.78
6bedclean	143270488	7	-6.8	10.365	2.108	2.987
6bedclean	143270488	8	-6.8	10.365	2.518	6.382
6bedclean	143270488	9	-6.7	12.271	2.721	6.896
6bedclean	143270488	10	-6.7	12.271	14.35	18.168
6bedclean	145293737	1	-8.3	0.8242	0	0
6bedclean	145293737	2	-8.3	0.8242	3.435	7.605
6bedclean	145293737	3	-8	1.3676	3.318	8.093
6bedclean	145293737	4	-8	1.3676	4.168	6.296
6bedclean	145293737	5	-8	1.3676	3.27	6.589
6bedclean	145293737	6	-7.9	1.6191	1.823	7.566
6bedclean	145293737	7	-7.8	1.9167	2.919	7.221
6bedclean	145293737	8	-7.7	2.2691	3.791	7.641
6bedclean	145293737	9	-7.7	2.2691	2.808	5.813
6bedclean	145293737	10	-7.6	2.6864	3.097	6.24
6bedclean	153640723	1	-8	1.3676	0	0
6bedclean	153640723	2	-7.9	1.6191	2.609	6.196
6bedclean	153640723	3	-7.8	1.9167	2.354	5.69
6bedclean	153640723	4	-7.8	1.9167	3.641	5.669
6bedclean	153640723	5	-7.7	2.2691	2.959	6.947
6bedclean	153640723	6	-7.6	2.6864	1.874	4.644
6bedclean	153640723	7	-7.2	5.2767	2.183	5.411
6bedclean	153640723	8	-7	7.3955	14.459	18.275
6bedclean	153640723	9	-7	7.3955	4.07	7.147
6bedclean	153640723	10	-6.9	8.7552	3.354	6.85
6bedclean	24848320	1	-7.4	3.765	0	0

6bedclean	24848320	2	-7.2	5.2767	3.331	4.958
6bedclean	24848320	3	-7.1	6.2469	3.503	4.447
6bedclean	24848320	4	-7.1	6.2469	0.943	6.525
6bedclean	24848320	5	-7.1	6.2469	3.015	4.583
6bedclean	24848320	6	-6.7	12.271	16.545	20.468
6bedclean	24848320	7	-6.6	14.527	17.372	20.619
6bedclean	24848320	8	-6.6	14.527	16.464	20.678
6bedclean	24848320	9	-6.5	17.198	15.496	20.106
6bedclean	24848320	10	-6.5	17.198	15.986	19.973
6bedclean	4212	1	-7.3	4.4572	0	0
6bedclean	4212	2	-7.3	4.4572	2.603	7.251
6bedclean	4212	3	-7.2	5.2767	1.124	6.384
6bedclean	4212	4	-7.2	5.2767	3.06	4.036
6bedclean	4212	5	-7	7.3955	3.432	5.108
6bedclean	4212	6	-6.9	8.7552	3.637	5.716
6bedclean	4212	7	-6.9	8.7552	3.621	7.007
6bedclean	4212	8	-6.7	12.271	1.538	5.963
6bedclean	4212	9	-6.7	12.271	3.873	4.892
6bedclean	4212	10	-6.5	17.198	3.178	6.282
6bedclean	44275839	1	-7.6	2.6864	0	0
6bedclean	44275839	2	-7.5	3.1803	3.804	7.801
6bedclean	44275839	3	-7.5	3.1803	3.083	6.283
6bedclean	44275839	4	-7.4	3.765	3.661	5.78
6bedclean	44275839	5	-7.4	3.765	3.154	6.166
6bedclean	44275839	6	-7.3	4.4572	3.895	7.898
6bedclean	44275839	7	-7.2	5.2767	4.509	7.249
6bedclean	44275839	8	-7.2	5.2767	3.346	7.68
6bedclean	44275839	9	-7	7.3955	2.998	5.69
6bedclean	44275839	10	-6.9	8.7552	0.894	6.483
6bedclean	44316536	1	-7.5	3.1803	0	0
6bedclean	44316536	2	-7.5	3.1803	3.74	6.891
6bedclean	44316536	3	-7.1	6.2469	3.453	7.286
6bedclean	44316536	4	-6.7	12.271	2.434	5.891
6bedclean	44316536	5	-6.7	12.271	2.902	3.806
6bedclean	44316536	6	-6.6	14.527	21.711	25.84
6bedclean	44316536	7	-6.6	14.527	24.983	29.06
6bedclean	44316536	8	-6.6	14.527	16.654	21.132
6bedclean	44316536	9	-6.6	14.527	23.64	27.402
6bedclean	44316536	10	-6.6	14.527	16.003	19.847
6bedclean	44541200	1	-7.4	3.765	0	0
6bedclean	44541200	2	-7.3	4.4572	1.23	5.99

6bedclean	44541200	3	-7.3	4.4572	1.724	2.171
6bedclean	44541200	4	-7.2	5.2767	3.002	5.938
6bedclean	44541200	5	-7	7.3955	3.806	7.1
6bedclean	44541200	6	-6.8	10.365	1.926	5.955
6bedclean	44541200	7	-6.8	10.365	3.54	7.498
6bedclean	44541200	8	-6.8	10.365	16.839	21.037
6bedclean	44541200	9	-6.7	12.271	16.685	20.584
6bedclean	44541200	10	-6.6	14.527	2.752	6.1
6bedclean	44541201	1	-7.5	3.1803	0	0
6bedclean	44541201	2	-7.3	4.4572	4.715	6.942
6bedclean	44541201	3	-7.2	5.2767	2.383	6.837
6bedclean	44541201	4	-6.9	8.7552	2.968	5.72
6bedclean	44541201	5	-6.8	10.365	3.139	5.258
6bedclean	44541201	6	-6.8	10.365	14.899	19.995
6bedclean	44541201	7	-6.8	10.365	3.302	4.49
6bedclean	44541201	8	-6.8	10.365	15.352	18.503
6bedclean	44541201	9	-6.7	12.271	4.312	9.247
6bedclean	44541201	10	-6.7	12.271	3.279	6.919
6bedclean	58102019	1	-7.5	3.1803	0	0
6bedclean	58102019	2	-7.4	3.765	0.709	6.205
6bedclean	58102019	3	-7.3	4.4572	3.374	5.007
6bedclean	58102019	4	-7.3	4.4572	3.958	7.268
6bedclean	58102019	5	-7.2	5.2767	3.753	5.568
6bedclean	58102019	6	-7	7.3955	2.002	5.499
6bedclean	58102019	7	-6.8	10.365	3.222	4.296
6bedclean	58102019	8	-6.8	10.365	3.138	7.577
6bedclean	58102019	9	-6.8	10.365	3.121	7.321
6bedclean	58102019	10	-6.7	12.271	2.678	7.373
6bedclean	59835539	1	-7.9	1.6191	0	0
6bedclean	59835539	2	-7.9	1.6191	3.584	6.711
6bedclean	59835539	3	-7.8	1.9167	3.841	5.79
6bedclean	59835539	4	-7.8	1.9167	1.714	7.477
6bedclean	59835539	5	-7.5	3.1803	3.769	7.848
6bedclean	59835539	6	-7.5	3.1803	3.073	6.723
6bedclean	59835539	7	-7.5	3.1803	2.57	5.6
6bedclean	59835539	8	-7.2	5.2767	3.284	7.463
6bedclean	59835539	9	-7.1	6.2469	3.093	8.038
6bedclean	59835539	10	-7.1	6.2469	3.009	6.589
6bedclean	71044822	1	-7.3	4.4572	0	0
6bedclean	71044822	2	-7.2	5.2767	2.978	7.105
6bedclean	71044822	3	-7	7.3955	1.779	2.188

6bedclean	71044822	4	-7	7.3955	2.478	4.251
6bedclean	71044822	5	-6.7	12.271	3.561	8.038
6bedclean	71044822	6	-6.7	12.271	3.663	5.498
6bedclean	71044822	7	-6.7	12.271	2.953	3.459
6bedclean	71044822	8	-6.5	17.198	3.67	4.389
6bedclean	71044822	9	-6.4	20.36	3.73	7.7
6bedclean	71044822	10	-6.3	24.103	17.168	21.518

Table S5: Pharmacophore models with generated scores.

**Pharmacophore Model**	**Score**
Model -1	0.9350
Model -2	0.9343
Model -3	0.9311
Model -4	0.9269
Model -5	0.9220
Model -6	0.9194
Model -7	0.9191
Model -8	0.9187
Model -9	0.9185
Model -10	0.9160
